# Accelerated brain aging predicts impaired cognitive performance and greater disability in geriatric but not midlife adult depression

**DOI:** 10.1038/s41398-020-01004-z

**Published:** 2020-09-18

**Authors:** Seth Christman, Camilo Bermudez, Lingyan Hao, Bennett A. Landman, Brian Boyd, Kimberly Albert, Neil Woodward, Sepideh Shokouhi, Jennifer Vega, Patricia Andrews, Warren D. Taylor

**Affiliations:** 1 Department of Psychiatry and Behavioral Sciences, Vanderbilt University Medical Center, Nashville, TN USA; 2 Department of Biomedical Engineering, Vanderbilt University, Nashville, TN USA; 3 Department of Electrical Engineering and Computer Science, Vanderbilt University, Nashville, TN USA; 4 Geriatric Research, Education, and Clinical Center, Veterans Affairs Tennessee Valley Health System, Nashville Tennessee, Nashville, TN USA

**Keywords:** Depression, Neuroscience

## Abstract

Depression is associated with markers of accelerated aging, but it is unclear how this relationship changes across the lifespan. We examined whether a brain-based measure of accelerated aging differed between depressed and never-depressed subjects across the adult lifespan and whether it was related to cognitive performance and disability. We applied a machine-learning approach that estimated brain age from structural MRI data in two depressed cohorts, respectively 170 midlife adults and 154 older adults enrolled in studies with common entry criteria. Both cohorts completed broad cognitive batteries and the older subgroup completed a disability assessment. The machine-learning model estimated brain age from MRI data, which was compared to chronological age to determine the brain–age gap (BAG; estimated age-chronological age). BAG did not differ between midlife depressed and nondepressed adults. Older depressed adults exhibited significantly higher BAG than nondepressed elders (Wald *χ*^2^ = 8.84, *p* = 0.0029), indicating a higher estimated brain age than chronological age. BAG was not associated with midlife cognitive performance. In the older cohort, higher BAG was associated with poorer episodic memory performance (Wald *χ*^2^ = 4.10, *p* = 0.0430) and, in the older depressed group alone, slower processing speed (Wald *χ*^2^ = 4.43, *p* = 0.0354). We also observed a statistical interaction where greater depressive symptom severity in context of higher BAG was associated with poorer executive function (Wald *χ*^2^ = 5.89, *p* = 0.0152) and working memory performance (Wald *χ*^2^ = 4.47, *p* = 0.0346). Increased BAG was associated with greater disability (Wald *χ*^2^ = 6.00, *p* = 0.0143). Unlike midlife depression, geriatric depression exhibits accelerated brain aging, which in turn is associated with cognitive and functional deficits.

## Introduction

Aging has an inevitable effect at molecular, cellular, and organ levels, with biological aging resulting in degeneration or reduction in the organ’s reparative or regenerative potential^1^. “Accelerated aging” refers to biological aging processes that occur more rapidly than expected, resulting in biological characteristics appearing older than expected based on the individual’s chronological age. Accelerated aging may result from numerous disease processes and can be quantified using a variety of markers such as oxidative stress measures^2^, telomere length^3^ or epigenetic measures of methylation such as Horvath’s epigenetic clock^4^. Differences in these markers of accelerated aging are reported in neuropsychiatric disorders including schizophrenia, post-traumatic stress disorder, anxiety disorders, and depression^5^. Depression is specifically associated with decreased telomere length^3^, while a multi-biomarker index of aging derived from measures of inflammation, metabolism, and organ function predicted greater depression severity in older adults^6^. Importantly, rates of aging as measured by biomarkers differ across organ systems. As the central nervous system may age differently than the rest of the body^7^, brain-specific markers of biological aging may be particularly germane to neuropsychiatric disorders.

By building on large databases of normative aging, examination of structural MRI data allows for examination of accelerated aging in the brain itself. One approach, the Brain Age Gap Estimation (BrainAGE) method^8^, utilizes a machine-learning technique for identifying individual-level variability in brain aging. Using standard structural MRI sequences, a prediction model generated from a learning sample of neurologically healthy adults can be applied to a new individual brain MRI to estimate that individual’s apparent biological age. The difference between this estimated biological age and the subject’s chronological age yields the brain-age “gap” (BAG), a marker of how much “older” or “younger” a given brain appears relative to the individual’s chronological age. This technique has been applied in psychiatric populations including schizophrenia, where particularly obese individuals exhibit older-appearing brains, although differences in estimated brain age are not seen in patients with bipolar disorder^9–11^. Past studies using brain age estimation techniques examining adult major depressive disorder (MDD) report that individuals with MDD tend to exhibit older estimated brain ages than expected^12^, although this finding is not universal^13^. If there is a difference in brain aging in MDD, it is possible it is associated with chronicity or recurrence of depressive episodes. This hypothesis is supported by studies associating greater chronicity or duration of depression with volumetric differences in key regions such as the hippocampus ^14–16^.

The examination of brain aging may have particular utility in older populations and late-life depression, or geriatric MDD. Geriatric MDD is associated with cerebrovascular pathology^17^, higher risk for dementia^18^, and greater medical morbidity^19^ that may contribute to impairment in multiple cognitive domains^20^ and accelerated brain aging. For example, diabetes mellitus, a risk factor both for cerebrovascular disease, dementia, and depression, is also associated with an increased brain-age gap^21^. The potential utility of brain-age estimation is supported by work in Alzheimer’s disease, where patients exhibit a greater brain-age gap than seen in cognitively intact elders^22^ and a greater discrepancy between estimated biological brain age and chronological age predicts conversion from mild cognitive impairment to dementia^23^. Structural MRI as a marker of accelerated aging may therefore provide new insights in the interactions between aging, depression, cognition, and disability.

In this study, we examined the BAG in two separate age cohorts, one of young- to midlife-adults and one of older adults. Both cohorts included both depressed and never-depressed subjects. The goal of this study was to determine, using BAG as a cross-sectional marker of accelerated aging, whether depressed individuals exhibited accelerated brain aging and if this measure is related to clinical outcomes, specifically cognitive performance, and disability. In primary analyses, we hypothesized that the estimated biological brain age of depressed participants would be older than their chronological age and that a greater BAG, indicating older biological age, would be associated with poorer cognitive performance and greater disability. In exploratory analyses in depressed subjects alone, we examined whether there were different relationships between BAG and cognition than was seen in the overall age cohorts, and whether greater depression severity modified the relationship between BAG and cognition.

## Methods

### Participants

The two age cohorts comprised one group of young-to-midlife adults with and without MDD (“adult cohort”) and another group of older adults with and without MDD (“geriatric cohort”). Other than the age criterion, these studies had similar entry criteria, with depressed participants being required to have a current diagnosis of MDD (DSM-IV-TR) and a Montgomery-Asberg Depression Rating Scale (MADRS)^24^ score of 15 or greater. The studies shared common exclusion criteria including acute suicidality, current or past psychosis, current psychotherapy, electroconvulsive therapy in the previous 6 months, presence of central neurological disease, diagnosis of dementia, or unstable medical conditions, developmental disorders, and MRI contraindications. Never-depressed participants had neither a history of psychiatric diagnoses nor a history of mental health treatment. Both age cohorts were outpatients recruited from clinical referrals and community advertisements.

The adult cohort was enrolled at Duke University Medical Center. The eligible age range was 20–50 years. For depressed participants, entry criteria further specified a diagnosis of recurrent MDD with the onset of a first depressive episode prior to age 35 years and no antidepressant medication use in the last month. Exclusion criteria included other lifetime DSM-IV Axis I disorders including substance abuse or dependence, Axis II disorders identified by the SCID-II^25^, use of illicit substances in the last month, a first-degree relative family history of bipolar disorder, or history of clinically relevant head injury.

The geriatric cohort was recruited at Vanderbilt University Medical Center as part of three separate studies with common entry criteria. Participants were age 60 years or older without a diagnosis of dementia or significant cognitive impairment assessed by a Montreal Cognitive Assessment (MoCA)^26^ score greater than 24 or a Mini Mental State Exam (MMSE)^27^ score greater than 24. For depressed participants, exclusion criteria included current or past Axis I disorder diagnoses, except for anxiety symptoms occurring during a depressive episode, history of substance abuse or dependence over the prior three years, and acute grief. Antidepressant medications were allowed in one geriatric study, with 9 of 14 depressed participants taking stable-dose antidepressant monotherapy at the time of MRI. The other two studies mandated no antidepressant use in the two weeks prior to MRI.

The Duke University Medical Center Institutional Review Board and the Vanderbilt University Institutional Review Board approved the studies conducted at each institution. All study participants provided written informed consent. Data from the adult cohort has previously been reported^28,29^ and we have also reported cognitive data from the geriatric cohort ^20^.

### Clinical assessments

For both studies, the DSM-IV-TR diagnosis of MDD was made using the Mini-International Neuropsychiatric Interview (MINI, version 5.0)^30^ and confirmed by an interview with a study psychiatrist. In all studies, participants were assessed for depression severity with the MADRS and medical burden with the geriatric Cumulative Illness Rating Scale (CIRS). In one but not the other two geriatric MDD studies, disability burden was measured using the World Health Organization Disability Schedule 2.0 (WHODAS 2.0) ^31^.

Using procedures similar to past reports^15,29^, we quantified age of initial depression onset and duration of depression using a life-charting approach in a detailed clinical interview, supplemented by acquisition of medical records. For adult MDD subjects, this was for lifetime duration of depression. For geriatric MDD subjects, this was limited to the current episode.

### Cognitive assessments

Participants completed a broad battery of neuropsychological tests that assessed cognitive domains relevant to depression or aging. As previously detailed^28,32,33^, we combined tests to create composite domain variables. We created *z*-scores for each measure based on the performance of all participants within each age cohort and averaged the *z*-scores for all tests within that domain. This resulted in a *z*-score for each domain for each participant. Cronbach’s alpha (CoA) was computed for each domain as a measure of internal consistency. As previously published^28^, for the adult cohort, tests in each domain included:

- Episodic Memory (CoA = 0.87): Logical Memory 1 and 2 from the Wechsler Memory Scale, Benton Visual Retention Test, Rey’s Verbal Learning Test (total I–V and total VII);
- Executive Function (CoA = 0.75): Controlled Oral Word Association (COWA) test (letters: C, F, L), Trail Making B time (reverse scored), semantic fluency (Animal Naming), Stroop Color-Word interference condition;
- Processing Speed (CoA = 0.70): Symbol-Digit Modality, Trail Making A time (reverse scored), Stroop Color Naming condition;
- Working Memory (CoA = 0.75): Digit Span forward and Digit Span backward from the Wechsler Memory Scale.

Two of the geriatric studies used identical neuropsychological test batteries and so were included in analyses examining cognitive performance. As previously published^20,33^, for the geriatric cohort, tests in each domain included:

- Episodic Memory (CoA = 0.75): Word List Memory Recall (immediate and delayed), Paragraph Recall test, Constructional Praxis test (delayed), Benton Visual Retention Test;
- Executive Function (CoA = 0.66): COWA test (letters: C, F, L), Trail Making B time (reverse scored), Stroop test color-word interference condition, Mattis Dementia Rating Scale, Initiation-Perseveration subscale;
- Processing Speed (CoA = 0.74): Symbol-Digit Modality Test, Trail-Making A time (reverse scored), Stroop color naming condition;
- Working Memory (CoA = 0.71): Digit Span forward, Digit Span backward, and Ascending Digits from the Wechsler Memory Scale.

### MRI acquisition

The adult cohort was imaged on a research-dedicated whole-body Siemens 3.0 T Trio Tim scanner at Duke University Medical Center using an 8-channel head coil. Parallel imaging was employed with an acceleration factor of 2. Duplicate sagittal MPRAGE sequences were obtained using a repetition time (TR) of 2300 ms, echo time (TE) of 3.46 ms, a flip angle of 9°, a 256 × 256 matrix, FOV 240 mm, 160 slices with a 1.2 mm slice thickness for voxel size of 0.9 × 0.9 × 1.2 mm.

The geriatric cohort was imaged on a research-dedicated 3.0 T Philips Achieva whole-body scanner at Vanderbilt University Medical Center using a 32-channel head coil. The MPRAGE images were obtained using TR = 8.75 ms, TE = 4.6 ms, flip angle = 9°, and spatial resolution = 0.89; × 0.89 × 1.2 mm^3^ plus a FLAIR T2-weighted imaging conducted with TR = 10,000 ms, TE = 125 ms, TI = 2700 ms, flip angle = 90°, and spatial resolution = 0.7 × 0.7 × 2.0 mm^3^. FLAIR T2-weighted imaging was also conducted using TR = 10,000 ms, TE = 125 ms, TI = 2,700 ms, flip angle = 90°, and spatial resolution = 0.7 × 0.7 × 2.0 mm^3^.

### MRI analyses and calculation of brain age

The brain age estimator is an automated deep learning tool used to predict or estimate age from a T1-weighted brain MRI. The first step in the brain age biomarker pipeline is to align the subject T1-weighted brain MRI with the MNI-305 template^34^ using the affine registration from the NiftiReg library^35^. Images also undergo N4 bias field correction^36^ to alleviate bias from acquisition. The input to the brain age estimation algorithm consists of the preprocessed brain MRI described above as well as the volume of 132 distinct regions of interest in the brain, obtained from a whole-brain segmentation using a multi-atlas technique^37^. The BrainAGE algorithm described by Bermudez et. al^8^. uses a deep convolutional neural network regression model trained on over 5000 healthy controls ages 4–96 to predict age with high accuracy^8^. The innovation presented from this work is the addition of anatomical context in the form of volumetric estimates of regions of interest throughout the brain, which resulted in a more accurate prediction of age. The output is the estimated age for that subject, with the BAG biomarker being the difference between chronological true age and algorithm estimated age. For this study, we conducted model inference in our two cohorts using the BAG algorithm without any further model optimization or changes. Brain age calculations were performed on an NVIDIA GeForce Titan GPU with 12 GB memory and all deep learning algorithms were implemented and tested using Tensorflow v1.4 with a Keras backend v2.2. In order to analyze this cohort, we used a large-scale medical image processing infrastructure^38^ and high performance computing cluster at Vanderbilt University. Trained models and analysis code for the BAG prediction used by Bermudez et al^8^. are publicly available at (https://github.com/MASILab/BrainAGE).

The Lesion Segmentation Toolbox^39^ was used to measure white matter hyperintensity (WMH) volumes, findings on T2- weighted FLAIR images related to cerebral ischemia. These analyses were implemented through the VBM8 toolbox in SPM8 and have been previously described^20,40^. This lesion map is then used to calculate total cerebral WMH volume. This process was only applied to the geriatric cohort data as the adult cohort imaging protocol did not include the necessary FLAIR sequence.

### Analytic plan

Statistical analyses were conducted using SAS Studio 3.8 (SAS Institute, Cary, NC). Participant demographics within each cohort were summarized and univariate comparisons conducted using pooled, two-tailed *t*-tests for continuous variables and chi-square tests for categorical variables. Data were graphed to facilitate the identification of outliers and one geriatric participant exhibited cognitive domain *z*-scores several standard deviations lower than the rest of the geriatric cohort. This individual was excluded from analyses.

The two age cohorts were not combined, and data were analyzed separately. As MRI data from each age cohort were gathered at different sites using different scanners (younger- to midlife adults at Duke, older adults at Vanderbilt), this created a fundamental confound between age and scanner type that cannot be clearly disentangled. Additionally, deep neural networks are unstable to inhomogeneities in medical imaging^41^, so scanner and site effects may introduce additional variability in the brain age estimation between study cohorts that could obfuscate or mask underlying biological variability.

**Fig. 1:**
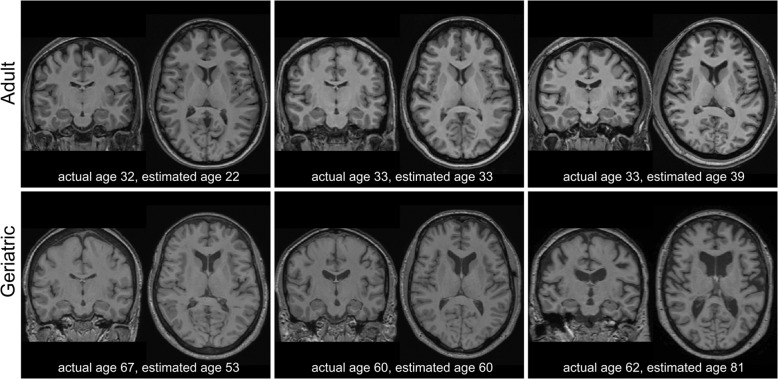
Comparison of structural MRI of participant brains in midlife and older adult cohorts. Each image is a separate participant, displaying coronal and axial images and actual (chronological) age and estimated (calculated) age. The top row is from the midlife adult cohort and the bottom row is from the geriatric cohort.

The primary imaging measure was the BAG, calculated as the difference between the algorithm-determined estimated age and the chronological age. A negative BAG indicated that the brain appeared younger than anticipated based on chronological age, while a positive BAG indicated a brain appearing older than anticipated (Fig. 1). As the BAG was calculated from but did not capture subjects’ chronological age, we included chronological age as a covariate in statistical models. This decision was based on age itself having a strong effect on brain structure, the importance of a given BAG value may vary based on chronological age, and because we hypothesized that there may be more variability in BAG with advancing age.

Primary analyses focused on testing for diagnostic group differences in BAG and the relationship between BAG, cognition, and disability. Statistical analyses used general linear models (PROC GENMOD) with a similar approach for both age cohorts. Initial models tested for diagnostic group differences in BAG, including covariates of chronological age, sex, education, and medical morbidity by CIRS. This was followed by examining the effect of BAG on z-scored cognitive domains, including covariates of the diagnostic group, chronological age, sex, education, and medical morbidity measured by CIRS. As an exploratory component of these analyses, we tested for a statistical interaction between diagnostic group and BAG affecting cognitive domain score. Similar approaches were used to assess the effects of BAG on disability measured by the WHODAS 2.0.

Subsequent exploratory analyses focused on the effects of depression history or depression severity, so included only depressed subjects. These models included covariates of chronological age, sex, education, CIRS, and depression severity by MADRS. We first tested for the relationship between BAG and depression exposure, examined both as the age of onset of the initial depressive episode and duration of depression, calculated as lifetime exposure for the adult MDD group and duration of the current episode for the geriatric MDD group. We next examined the effect of BAG on z-scored cognitive domain performance in the depressed groups alone, also testing for statistical interaction between depression severity and BAG.

As WMHs are common in geriatric MDD, associated with aging^42,43^, and a potential marker of accelerated cerebrovascular aging, in further exploratory analyses we examined the relationship between WMH volume, BAG, and clinical measures. As WMH volumes are often not normally distributed, our primary measure was log-transformed WMH volume. These exploratory analyses examined the same models as detailed above but included transformed WMH volume as an additional covariate. These were only conducted in the geriatric MDD cohort data as WMH are uncommon outside the geriatric age range and the adult cohort did not have the required FLAIR MRI acquisition.

## Results

### Brain age analyses in the midlife adult cohort

The adult cohort included 76 depressed and 94 non-depressed adults (Table 1). Depressed participants were significantly older than never-depressed participants in chronological age and estimated age but exhibited a comparable BAG. Depressed participants exhibited significantly higher medical comorbidity severity (via CIRS) and, in univariate analyses, poorer episodic memory, and processing speed performance. The depressed subjects’ mean age of onset was 20.8 years (range 6–35), with a mean lifetime depression duration of 2115 days (5.8 years; range 90–7500 days).

**Table 1:** Demographic and clinical differences across samples.

	Adult sample				Geriatric sample			
	Depressed (*N* = 76)	Never-depressed (*N* = 94)	Test value	*p*-value	Depressed (*N* = 118)	Never-depressed (*N* = 36)	Test value	*p*-value
Age, years (chronologic)	36.21 (9.04)	30.14 (9.2)	4.30	<0.0001	66.41 (5.45)	70.06 (6.65)	3.33	0.0011
Age, years (estimated)	43.67 (11.27)	37.48 (10.09)	3.77	0.0002	70.09 (8.12)	68.83 (10.59)	0.66	0.5117
Brain-age gap (BAG)	7.46 (7.56)	7.33 (5.54)	0.12	0.9027	3.69 (7.16)	−1.23 (7.62)	3.55	0.0005
Sex, % female (*N*)	68.42% (52/76)	61.70% (58/94)	0.83	0.3621	61.0 (72/118)	55.6 (20/36)	0.34	0.5586
Race, % minority (*N*)	40.79% (31/76)	50.00% (47/94)	1.43	0.2308	10.17% (12/118)	11.11% (4/36)	0.02	0.8712
Education, years	15.34 (2.43)	15.69 (2.06)	1.01	0.3123	16.79 (2.21)	17.17 (1.93)	0.92	0.3573
CIRS	0.67 (1.16)	0.31 (0.75)	2.36	0.0199	5.45 (3.22)	4.72 (2.77)	1.22	0.2234
MADRS	23.62 (4.33)	0.80 (1.16)	44.7	<0.0001	26.21 (5.20)	0.75 (1.02)	50.11	<0.0001
WMH volume (log)	–	–			0.18 (1.48)	0.13 (1.43)	0.14	0.8894
					*N* = 103	*N* = 36		
MMSE	–	–			29.20 (1.1)	29.3 (1.1)	0.23	0.8186
Episodic memory	−0.68 (4.24)	0.85 (3.68)	2.49	0.0137	−0.03 (0.73)	0.15 (0.74)	1.26	0.2104
Executive function	−0.36 (3.05)	0.46 (2.92)	1.79	0.0754	−0.08 (0.69)	0.29 (0.51)	3.37	0.0012
Processing speed	−0.51(2.49)	0.51 (2.20)	2.82	0.0053	−0.05 (0.72)	0.30 (0.54)	3.03	0.0033
Working memory	−0.21 (1.67)	0.20 (1.88)	1.49	0.1370	−0.02 (0.82)	0.15 (0.81)	1.12	0.2632
					*N* = 85	*N* = 15		
WHODAS	–	–			23.91 (14.82)	4.51 (4.05)	10.21	<0.0001

Data presented as mean (standard deviation) for continuous variables and percent (*N*) for categorical variables. Analyses used pooled, two-tailed *t*-tests for continuous variables and chi-square tests with 1 df for categorical variables. Pooled *t*-tests for the adult sample had 168 degrees of freedom. In pooled *t*-tests for the geriatric sample, for the overall demographics df = 152, for the cognition sample df = 137, and for the disability sample df = 98. The exceptions requiring the use of Satterthwaite t-tests due to unequal variances for the adult sample included analyses of BAG (133.8 df), (CIRS (122.8 df) and MADRS (83.7 df), and for the geriatric sample analyses of MADRS (141.05 df), estimated age (48.2 df), executive function (81.1 df), processing speed (87.7 df), and WHODAS score (82.0 df).

*BAG* brain-age gap, *CIRS* Cumulative Illness Rating Scale, *MADRS* Montgomery-Asberg Depression Rating Scale, *MMSE* mini-mental state examination, *WHODAS* World Health Organization Disability Assessment Schedule (Version 2.0), presented as percent disabled, *WMH* white matter hyperintensity.

After controlling for covariates (chronological age, sex, education, CIRS) BAG did not differ between depressed and non-depressed participants (Wald *Χ*^2^ = 0.40, 164 df, *p* = 0.5294). BAG was also not significantly associated with episodic memory (Wald *Χ*^2^ = 1.56, 163 df, *p* = 0.2112), executive function (Wald *Χ*^2^ = 1.94, 163 df, *p* = 0.1637), processing speed (Wald *Χ*^2^ = 0.01, 163 df, *p* = 0.9210), or working memory (Wald *Χ*^2^ = 0.02, 163 df, *p* = 0.8837.) Tests for an interactive effect between MDD diagnosis and BAG on cognitive performance were not statistically significant, thus the relationship between BAG and cognitive performance did not appear to differ based on a diagnosis of MDD (data not shown).

In exploratory analyses of depressed participants only, depression severity by MADRS was not significantly associated with BAG (Wald *Χ*^2^ = 0.25, 70 df, *p* = 0.6141). We further did not observe significant relationships between BAG and age of onset (Wald *Χ*^2^ = 0.06, 68 df, *p* = 0.8098) or lifetime duration of depression (Wald *Χ*^2^ = 0.35, 68 df, *p* = 0.5515). In depressed participants alone, there were neither significant direct effects of BAG nor interactive effects between MADRS and BAG on cognitive domain performance (data not shown).

### Brain age analyses in the geriatric cohort

The geriatric cohort included 118 depressed and 36 never-depressed elders (Table 1). Compared to non-depressed participants, depressed elders were younger with a lower mean chronological age but exhibiting comparable estimated age. This resulted in depressed elders exhibiting a significantly higher BAG. Depressed elders exhibited poorer performance on unadjusted measures of executive function and processing speed. For depressed elders, the mean age of onset was 34.7 years (range 5–84 years), with a mean current episode duration of 953 days (2.6 years, range 15–5141 days).

**Fig. 2:**
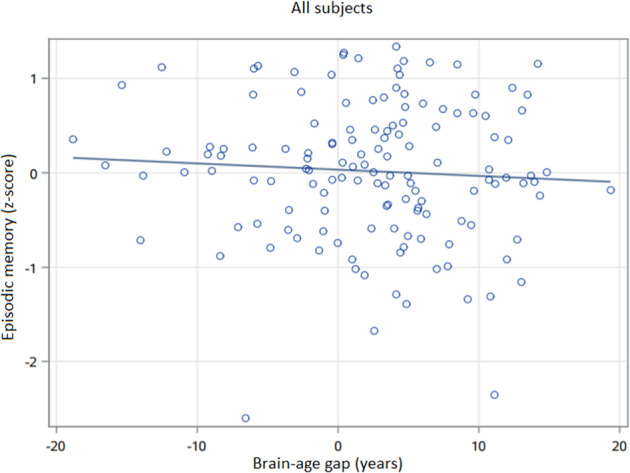
Association between brain-age gap and episodic memory in geriatric subjects. BAG was associated with episodic memory in general linear models (Wald *Χ*^2^ = 4.10, 132 df, *p* = 0.0430), including covariates of diagnosis, age, sex, education, and medical morbidity. Episodic memory has no units, presented as an average *z*-score across tests. Brain-age gap (BAG) is in years, calculated as the difference between the calculated estimated age and the chronological age.

**Fig. 3:**
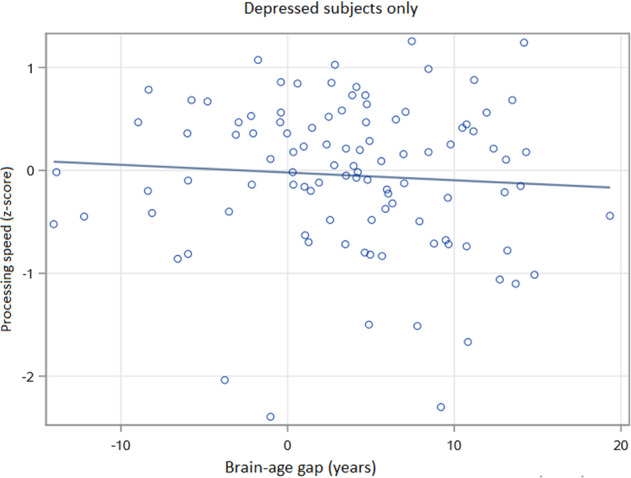
Association between brain-age gap and disability (WHODAS) in older adults. BAG was associated with disability in general linear models (Wald *Χ*^2^ = 6.00, 93 df, *p* = 0.0143), including covariates of diagnosis, age, sex, education, and medical morbidity. Disability measured by the WHODAS 2.0, calculated as percent disabled. Brain-age gap (BAG) is in years, calculated as the difference between the calculated estimated age and the chronological age.

After adjusting for chronological age, sex, education, and CIRS, BAG was significantly higher in depressed elders (Wald *Χ*^2^ = 8.84, 148 df, *p* = 0.0029) indicating that the brains of depressed participants appeared older than expected by chronological age alone. After adjusting for covariates including diagnosis, a higher BAG was associated with poorer episodic memory performance (Wald *Χ*^2^ = 4.10, 132 df, *p* = 0.0430; Fig. 2) but not executive function (Wald *Χ*^2^ = 0.03, 132 df, *p* = 0.8643), processing speed (Wald *Χ*^2^ = 2.78, 132 df, *p* = 0.0957), or working memory (Wald *Χ*^2^ = 0.00, 132 df, *p* = 0.9974). Tests for an interactive effect between MDD diagnosis and BAG on cognitive performance were not statistically significant (data not shown). Disability data measured by WHODAS was available from one study, consisting of data from 85 depressed and 15 never-depressed older participants. After adjusting for covariates, BAG was associated with greater disability (Wald *Χ*^2^ = 6.00, 93 df, *p* = 0.0143; Fig. 3).

**Fig. 4:**
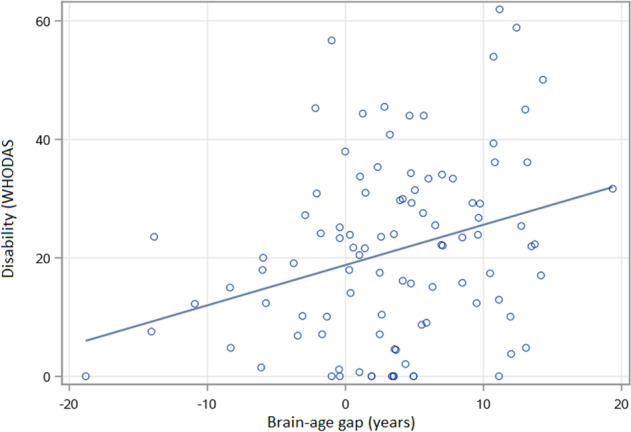
Association between brain-age gap and processing speed in older depressed subjects. BAG was associated with processing speed in depressed elders in general linear models (Wald *Χ*^2^ = 4.43, 96 df, *p* = 0.0354), including covariates of depression severity (MADRS), age, sex, education, and medical morbidity. Processing speed has no units, presented as an average *z*-score across tests. Brain-age gap (BAG) is in years, calculated as the difference between the calculated estimated age and the chronological age.

In exploratory analyses examining depressed elders only, there was no statistically significant relationship between BAG and depression severity by MADRS (Wald *Χ*^2^ = 0.96, 112 df, *p* = 0.3271). We also did not observe significant relationships between BAG and either age of onset (Wald *Χ*^2^ = 0.31, 110 df, *p* = 0.5769), or duration of current episode (Wald *Χ*^2^ = 0.05, 110 df, *p* = 0.8225). In models examining depressed elders only (*N* = 103), controlling for age, sex, education, CIRS and MADRS, there was a primary effect of BAG on processing speed (Wald *Χ*^2^ = 4.43, 96 df, *p* = 0.0354; Fig. 4) but not episodic memory (Wald *Χ*^2^ = 1.07, 96 df, *p* = 0.2999), executive function (Wald *Χ*^2^ = 0.30, 96 df, *p* = 0.5836), or working memory (Wald *Χ*^2^ = 1.00, 96 df, *p* = 0.3164). We further observed statistically significant interactive effects between MADRS and BAG on executive function (Wald *Χ*^2^ = 5.89, 95 df, *p* = 0.0152) and working memory (Wald *Χ*^2^ = 4.47, 95 df, *p* = 0.0346). In these analyses, a greater BAG had an increasingly negative effect on executive function and working memory in context of worsening MADRS score. In other words, the effect of a higher BAG (or having an older-appearing brain than expected) on executive function and working memory performance is greater in context of more severe depressive symptoms. No significant interaction effects were observed between MADRS and BAG on episodic memory (Wald *Χ*^2^ = 0.98, 95 df, *p* = 0.3213) or processing speed (Wald *Χ*^2^ = 0.04, 95 df, *p* = 0.8448).

### Effect of WMH on the BAG and its clinical correlates in geriatric MDD

As greater severity of WMH is an aging-related marker of vascular damage in geriatric MDD, in further exploratory analyses we examined the relationship between log-transformed WMH volume, BAG, and diagnosis. When adding log-transformed WMH volume to the models described above, WMH was positively associated with BAG (Wald *Χ*^2^ = 7.01, 147 df, *p* = 0.0081), but this did not appreciably change the relationship between MDD diagnosis and BAG (Wald *Χ*^2^ = 7.00, 147 df, *p* = 0.0082).

We next examined whether the addition of log-transformed WMH volume to models examining cognitive performance and disability changed the results described above. The addition of WMH did not appreciably change our observed associations between BAG, cognitive performance, or disability (data not shown), except for the interactive effect observed in the depressed subjects between BAG and MADRS on executive function. Despite WMH not being significantly associated with executive function in this model (Wald *Χ*^2^ = 0.99, 94 df, *p* = 0.3202), the interaction between BAG and MADRS was also no longer statistically significant (Wald *Χ*^2^ = 0.28, 94 df, *p* = 0.5979).

## Discussion

Differences in brain aging can be observed comparing chronological age with estimated calculated age using the BAG metric (Fig. 1, video displaying geriatric MRI data with both chronological and estimated ages at: https://vimeo.com/393048773). We observed marked differences in the clinical implications of the difference between estimated brain age and chronological age between younger and older cohorts. In the young to the middle-aged adult cohort, BAG did not differ by diagnosis and we observed no significant relationships between BAG and cognition. In contrast, depressed elders exhibited a higher BAG, indicating that the estimated age based on structural MRI was higher than expected. This higher BAG was associated with poorer episodic memory performance and greater disability. In exploratory analyses of depressed elders only, higher BAG was further associated with slower processing speed plus BAG exhibited an interactive effect with depression severity on executive function and working memory performance. Greater WMH volume, an MRI marker of pathological brain aging associated with geriatric depression^42,43^, was associated with a higher BAG, but largely did not change observed relationships between BAG and cognition or disability.

Our findings in adult MDD are generally concordant with some past work in this population^13^, suggesting that accelerated brain aging is not prominent in midlife adults even with recurrent depressive episodes. We now extend this approach to older adults. To our knowledge, this is among the first reports to use a deep learning approach to examine a brain-based biomarker of accelerated aging in geriatric MDD. The different findings observed in the two age cohorts raise the possibility that at some point depressed subjects may diverge from never-depressed individuals and the aging process accelerates, although due to technical limitations between the two age cohorts mean we cannot test this hypothesis with these data. If this hypothesis is correct, it is then unclear whether the acceleration of brain might be linear or if depressed and nondepressed groups diverge in a specific midlife window. Relevant to this hypothesis, we did not associate longer depression exposure with increased BAG. However, our findings may be constrained by using retrospective measures to assess duration of depression that are based primarily on remote patient recall.

In geriatric MDD, BAG is associated with cognition and disability. The association between increased BAG and poorer episodic memory is consistent with work showing episodic memory is particularly vulnerable even to normal aging^44^. Episodic memory is the hallmark domain affected in mild cognitive impairment and shows steeper decline in pathological aging such as in Alzheimer’s dementia. Similarly, other biomarkers of accelerated aging are associated with worse episodic memory performance in neuropsychiatric populations, including decreased telomere length and gray matter volume^45^. Conversely, in older adults, lower epigenetic age calculated from DNA methylation was predictive of intact episodic memory ^46^.

In exploratory analyses examining only older depressed subjects, we observed further associations between BAG and cognitive performance. The effect of BAG on processing speed in depressed elders is consistent with past reports of decreased processing speed in geriatric MDD^47,48^ that may serve as a core cognitive impairment, mediating deficits in working memory and verbal capabilities^47,48^. Intriguingly, we found an interaction between increased BAG and depression severity on executive function and working memory, although the effect on executive function did not persist after adjusting for WMH volume. It is well established that active depressive symptoms can worsen cognitive performance, even with treatment^49^. Our current findings suggest that the effect of accelerated brain aging on executive function and working memory may be mediated by depression severity. In other words, an individual with an older-appearing brain may exhibit a greater decline in these cognitive domains as their depression severity worsens. This may help explain variability in executive processes in late-life depression, and furthermore why performance may improve with successful antidepressant treatment. Such a “two-hit” hypothesis of an older-looking brain becoming increasingly vulnerable to the cognitive effects of a depressive episode is consistent with clinical experience. However, this interpretation should be viewed cautiously, and replication is needed as these were exploratory analyses in a subsample of the larger study.

Our observed association of higher BAG with a greater disability is supported by past work in older adults. Repeatedly, biomarkers of accelerated brain aging in both gray matter and the white matter are associated with increased disability and impairment in activities of daily livings. These findings include associations between disability and hippocampal volume loss, cortical gray matter changes, WMH, infarcts, and other measures of white matter microstructure^50–52^. Our observation utilizing a BAG measure derived from structural differences is concordant with this past work. It also identifies biological contributors to disability in geriatric MDD that extend beyond the severity of depressive symptoms.

We did not observe a significant relationship in either age cohort between BAG and depression exposure, either measured as age at onset of the initial depressive episode or duration of depressive episodes. This important negative finding was contrary to our hypothesis, as past work has associated depression exposure with other brain measures, most notably hippocampal volume^15,16,53^. Age of onset differences is also reported in measures of cortical thickness in frontal, temporal, and cingulate regions^54^. If we assume that cumulative depression exposure does contribute to volumetric brain changes, it is possible that more subtle, focal effects could not be detected using a brain-wide measure of estimated age. Alternatively, perhaps in some depressed individuals, repeated depressive episodes do not contribute to structural brain alterations. In either case, the recall bias inherent in retrospectively recalling depressive episodes contributes to inaccuracies in those measures, challenging our ability to definitively answer such questions in the absence of prospective studies.

A strength of the study includes two large cohorts across the adult lifespan. Limitations include the examination of cross-sectional rather than longitudinal data, limiting our ability to make causal inferences or determine whether BAG measures may have prognostic utility in predicting cognitive decline or worsening disability. We additionally did not have the ability to look at potentially protective behaviors that may be positively associated with a negative BAG, where brain age is younger than expected, such as physical activity or aerobic exercise. Moreover, our analytic plan included numerous comparisons, raising the potential for a Type 1 error. Given the novel nature of the analyses, we did not a priori plan to adjust for multiple comparisons. However, we did work to reduce the number of comparisons, such as examining z-transformed cognitive domain scores rather than examine performance across individual neuropsychological tests. Had we adjusted for multiple comparisons in the primary geriatric cohort analyses (using either a Bonferroni or False Discovery Rate approach), only the diagnostic group difference in the BAG would have retained statistical significance. This issue is also relevant for the exploratory analyses. Thus our findings should be viewed cautiously and warrant replication.

Although the size of our cohorts and the age range examined is a strength, an additional limitation is that we could not combine neuroimaging data across cohorts. As the two age cohorts were scanned exclusively on two different scanners, age is fundamentally confounded with scanner type. As deep neural networks are unstable to inhomogeneities in medical imaging^41^, combining data would lead to scanner effects that would introduce additional variability in the brain age estimation between study cohorts that could mask biological variability. Observed differences in brain age prediction between diagnostic groups are thus likely to be due to clinical differences instead of bias due to site effect, despite reducing the possible sample size if both cohorts were analyzed together. In addition, while our participants include younger and older adults, we have an age gap between the studies, with no participants between the ages of 50 and 59 years. These factors do not allow us to directly examine the effects of BAG across the entire adult age range and complicate our ability to test whether depression results in a divergence in estimated brain age from chronological age. These issues are highlighted by differences in the accuracy in age prediction between the two cohorts. We saw that the average BAG in the midlife diagnostic groups ~7 years, whereas the average BAG in the geriatric cohort was 3.7 years for depressed and −1.2 years for never-depressed subjects (Table 1). The model of age prediction used in this work relies on patterns in image intensity from the T1-weighted brain MRI to predict age. Deep neural networks are susceptible to small, systemic changes in image intensity^41^, so site or scanner effects, such as the differences between the two cohorts in this study, may bias age prediction.

Future work should continue to examine the clinical significance of measuring BAG and whether BAG may be predictive of short- or long-term outcomes such as acute antidepressant response or risk of recurrence following remission^55^. It should also be determined whether it has long-term prognostic value to identify individuals at increased risk of cognitive decline. Such work would be valuable in geriatric MDD but also early in the course of neurodegenerative disorders such as Alzheimer’s disease. This longitudinal work should examine BAG not only as a cross-sectional predictor, but also how BAG changes with aging, and whether such change trajectories may be more informative than cross-sectional assessments alone. Although this study does not support brain age as a clinically useful marker of accelerated aging in midlife adults with MDD, it does support a potential role for disorders of aging.
